# Musical Aptitude Is Associated with AVPR1A-Haplotypes

**DOI:** 10.1371/journal.pone.0005534

**Published:** 2009-05-20

**Authors:** Liisa T. Ukkola, Päivi Onkamo, Pirre Raijas, Kai Karma, Irma Järvelä

**Affiliations:** 1 Department of Medical Genetics, University of Helsinki, Helsinki, Finland; 2 Department of Biological and Environmental Sciences, University of Helsinki, Helsinki, Finland; 3 Sibelius Academy, DocMus Department, Helsinki, Finland; 4 Sibelius Academy, Department of Music Education, Helsinki, Finland; 5 Laboratory of Molecular Genetics, Helsinki University Central Hospital, Helsinki, Finland; University of Wuerzburg, Germany

## Abstract

Artistic creativity forms the basis of music culture and music industry. Composing, improvising and arranging music are complex creative functions of the human brain, which biological value remains unknown. We hypothesized that practicing music is social communication that needs musical aptitude and even creativity in music. In order to understand the neurobiological basis of music in human evolution and communication we analyzed polymorphisms of the arginine vasopressin receptor 1A (AVPR1A), serotonin transporter (SLC6A4), catecol-O-methyltranferase (COMT), dopamin receptor D2 (DRD2) and tyrosine hydroxylase 1 (TPH1), genes associated with social bonding and cognitive functions in 19 Finnish families (n = 343 members) with professional musicians and/or active amateurs. All family members were tested for musical aptitude using the auditory structuring ability test (Karma Music test; KMT) and Carl Seashores tests for pitch (SP) and for time (ST). Data on creativity in music (composing, improvising and/or arranging music) was surveyed using a web-based questionnaire. Here we show for the first time that creative functions in music have a strong genetic component (h^2^ = .84; composing h^2^ = .40; arranging h^2^ = .46; improvising h^2^ = .62) in Finnish multigenerational families. We also show that high music test scores are significantly associated with creative functions in music (p<.0001). We discovered an overall haplotype association with AVPR1A gene (markers RS1 and RS3) and KMT (p = 0.0008; corrected p = 0.00002), SP (p = 0.0261; corrected p = 0.0072) and combined music test scores (COMB) (p = 0.0056; corrected p = 0.0006). AVPR1A haplotype AVR+RS1 further suggested a positive association with ST (p = 0.0038; corrected p = 0.00184) and COMB (p = 0.0083; corrected p = 0.0040) using haplotype-based association test HBAT. The results suggest that the neurobiology of music perception and production is likely to be related to the pathways affecting intrinsic attachment behavior.

## Introduction

Composing and interpreting music by singing, playing an instrument or dancing are complex creative functions of the human brain, whose biological basis remains unknown [1]. Creativity and divergent thinking are sometimes considered as divisions of intelligence, suggesting creative functions may also have a genetic liability [2]. Although there is thus far little evidence for the biological underpinnings of creativity, the well-known child prodigy phenomenon in the music field suggests that genetic differences in musical creativity do exist [3]. Mere practice, environmental components (e.g., parental support) or chance are not enough to explain the exceptional creative achievements of Mozart, Yehudi Menuhin or Jacqueline du Pré at a very young age.

Composing, improvising and arranging music are high-level creative functions and defined as “creativity in music” in this article. Creativity is an ability to produce work that is both original and appropriate for the situation in which it occurs [4]. Creative activity varies in degree from individual small-scale creative insights to large-scale creative productivity with societal and economic aspects. Psychologically the creative inspiration arises in the state of mind where attention is activated [5]. Biologically it demands low levels of cortical activation, comparatively more right- than left-hemisphere activation, and low levels of frontal-lobe activation [6]. Furthermore, creativity requires the simultaneous presence of several traits, e.g. intelligence, perseverance, unconventionality and the ability to think in a particular manner [5]. A musician needs among others these traits when composing, improvising or arranging music. Bengtsson et al. [7] reported that the pianist's cortical regions such as the right dorsolateral prefrontal cortex, the pre-supplementary motor area, the rostral portion of the dorsal premotor cortex, and the left posterior part of the superior temporal gyrus were activated while improvising. More recently, prefrontal activity accompanied by widespread activation of neocortical sensory-motor areas was demonstrated in MRI studies of improvising professional jazz pianists [8]. However, research into creativity in music has been scarce up till now. Although some researchers (e.g. Gagné [9]) question whether a musician needs any creativity for playing an instrument or singing, they definitely agree that composing or improvising music is based on a musician's creativity.

Music perception and musical aptitude are cognitive functions of the human brain. In humans as well as other mammals the hormone arginine vasopressin (AVP) has a prominent role in controlling higher cognitive functions, such as memory and learning [10]. The AVP receptor 1A, that is coded by the AVPR receptor 1A gene, mediates the influences of the AVP hormone in the brain [11]. Additionally AVP has been shown to affect many social, emotional and behavioral traits, including pair bonding and aggression in males [12], [13], parenting [14], sibling relationships [15], love [16] and altruism [17].

The dopaminergic and serotoninergic system, and related genes, have been shown to influence cognitive and motor functions in human and animal studies [2], [18], [19]. The human serotonin transporter (SLC6A4; 5-HTT) is expressed in the brain, mainly in areas involved with emotions in the cortex and limbic systems. The role of the SLC6A4 polymorphism 5-HTTLPR has previously been studied in reward-seeking behaviors [20], and in emotional disorders [21], [22]. SLC6A4 together with arginine vasopressin receptor gene (AVPR1A) polymorphisms have been reported to associate with artistic creativity in professional dancers [23] and with short-term musical memory [24]. Tryptophan hydroxylase (TPH) is a rate-limiting enzyme in the biosynthesis of serotonin (5-HT), regulating the amount of serotonin available in the synaptic cleft [25]. Tryptophan hydroxylase gene 1 (TPH1) is responsible for peripheral serotonin generation [26]. TPH1 polymorphism A779C A-allele is associated with figural and numeric creativity [2]. Additionally TPH1 A779C has been associated to addiction [27].

Catechol-O-methyltransferase (COMT) is a critical enzyme involved in the degradation of dopamine [28]. COMT works by inactivating dopamine and other catecholamine neurotransmitters in the synaptic cleft. Val158Met polymorphism of the COMT gene increases COMT activity [29]. Carriers of the Val allele have been shown to have 40% higher COMT activity than those with the Met allele. Thus, Met allele carriers may have a cognitive advantage [30], [31]. Val158Met polymorphism has been related to basal cognitive processes. The low activity allele Met has been associated with memory [18], [32], [33], experience of reward [34] intelligence [35], and the high activity allele Val with emotional difficulties and addiction [36].

The role of dopamine receptor D2 gene (DRD2) has been studied in conjunction with several cognitive processes, including intelligence [2], [28], [35], [37], [38], learning from errors [39], and creativity in humans [2]. The DRD2 polymorphism TAQIA has two alleles named A1 and A2. The carriers of the A1 allele (denoted by A1+) have D2 dopamine receptor density reduced up to 30–40% compared to A1- [28].

We hypothesize that producing music by composing, improvising or arranging require an extremely complex network of cognitive processes; human emotional facets, creative thinking and musical aptitude. Here we analyzed whether the polymorphisms in the aforementioned five genes are associated with musical aptitude and creative functions in music in the Finnish multigenerational families with professional musicians and/or active amateurs.

## Materials and Methods

### Family material

**Figure 1:**
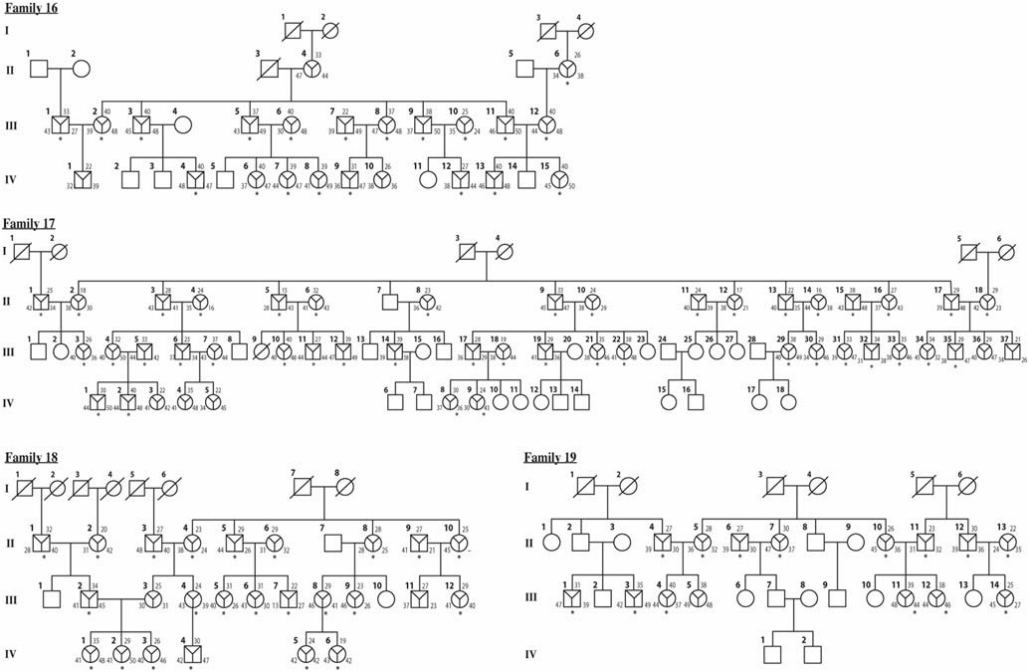
The pedigrees 16–19 participating in the study. Upper triangle, test score for KMT; left, test score for ST; right, test score for SP. Subjects who had given DNA for the genome-wide scan are marked by an asterisk (*).

A total of 19 Finnish families with 343 family members (150 males and 193 females) with at least some professional musicians and/or active amateurs participated in the music tests (Figure 1). The ages of the participants varied between 9 and 93 years (43 years mean age). DNA was obtained from 298 (86.9%) individuals over twelve years of age. The first 15 families have been described earlier [40]. The four new families were collected as described earlier [40] and are shown in Fig. 1.

The families were recruited for the study via a nationwide search by sending information leaflets or letters to the families whose members had studied/were studying at Sibelius Academy or other music institutes in Finland. The family members who first contacted us acted as a contact person to the other family members and informed them about the study. After that a testing session lasting about 1 hour (1–20 participant/session) was agreed with the family members interested in the study. In the beginning of the session the purpose of the study was explained to the participants by one of the authors (K.K., P.R or L.U). After verbal informed consent the three tests of musical aptitude were performed and a peripheral venous blood sample was collected for the study. The study was approved by The Ethical Committee of Helsinki University Central Hospital. Informed consent was obtained from all participating subjects.

### Tests for musical aptitude

The musical aptitude was assessed using three music tests: the auditory structuring ability test (Karma Music test, KMT) designed by one of the authors [41] and Carl Seashore's pitch and time discrimination subtests (SP and ST respectively) [42] as described in detail by Pulli et al. [40]. The test scores were shown to be heritable [40]. The KMT is designed to measure auditory structuring in a way that it should minimize the effects of training and/or culture [41]. In the KMT small, abstract sound patterns are repeated to form hierarchic structures. The subject's task is to detect structural changes in these patterns, i.e., changes in the order or number of the tones. The main components found in factor analyses of test data are grouping according to good gestalts, forming expectations, breaking gestalts, and changing expectations. The two last can be combined into flexibility of structuring or field independence [43]. KMT measures recognition of melodic contour, grouping, relational pitch processing, and gestalt principles, the same potentially innate musical cognitive operations reported by Justus & Hutsler [44]. In contrast, the Seashore's tests measure simple sensory capacities, such as the ability to detect small differences in tone pitch or duration that are necessary in music perception. Although there may be some overlap, the three tests used in this study mainly measure different parts of musical aptitude.

### Measuring creative functions in music

An extensive web-based self-report on-line questionnaire was designed. One part of the questionnaire was devised to chart the creative functions of the participants in music. An invitation letter was sent to participants by e-mail (if available) or traditional mail. The letter contained information about the research, the URL (Uniform Resource Locator) to the web site at University of Helsinki where the questionnaire was accessible and the instructions how to open the web site and answer the questions. It was also possible to ask for a paper-based questionnaire. Parents answered the questions on behalf of their children who were younger than 12 years of age. Creative functions were defined in this study as composing, improvising and/or arranging music. The participants were asked if they 1) compose music; 2) improvise music and/or 3) arrange music. Additionally, more detailed information (e.g. music education, musical training in general) was asked to confirm the answers on creativity in music in each participant.

### Genotyping

Peripheral venous blood samples were collected from the study subjects over 12 years of age, and DNA was extracted using the phenol-chloroform method.

### AVPR1A

We analyzed the highly variable microsatellites RS1 and RS3 residing in the promoter region and the AVR microsatellite in the intron of the AVPR1a gene [45]. The primers are shown in Table 1. The completion rates of the AVPR1A microsatellites ranged from 91% to 93%. Allele frequencies of RS1 and RS3 were in line with the studies of Bachner-Melman et al. [15], [23], and the study of Yirmiya et al. [46] (Table 2). RS1 allele 1 was not found in our study, and in the previous studies the allele was also rare (frequency 0.0218–0.0028). The prevalent alleles of RS1 in our study as in the aforementioned studies were alleles 3 and 4. In our study the prevalent allele of RS3 was allele 5, and in the studies of Bachner-Melman et al. [15], [23,], and Yirmiya et al. [46] it was allele 4. In the AVR microsatellite locus the most common allele was the same as reported by Yirmiya et al. [46]. AVPR1A microsatellites were run on an ABI 3730xl DNA Analyzer (Applied Biosystems, Foster City, CA, USA) and sized with GeneMapper 4.0 software (Applied Biosystems).

**Table 1:** Primers and conditions used.

Gene	Region	Forward primer	Reverse primer	TA (°C)
AVPR1a AVR	12q14-15, intron	5′-FAM-ATC CCA TGT CCG TCT GGA C-3′	5′-AGT GTT CCT CCA AGG TGC G-3′	60
AVPR1a RS1	12q14-15, promoter	5′-HEX-AGG GAC TGG TTC TAC AAT CTG C-3′	5′-ACC TCT CAA GTT ATG TTG GTG G-3′	60
AVPR1a RS3	12q14-15, promoter	5′-FAM-CCT GTA GAG ATG TAA GTG CT-3′	5′-TCT GGA AGA GAC TTA GAT GG-3′	60
SLC6A4 VNTR	17q, Intron 2	5′-FAM-TCAGTATCACAGGCTGCGAG-3′	5′-TGTTCCTAGTCTTACGCCAGTG-3′	58
SLC6A4 5-HTTLPR	Promoter	5′-GGCGTTGCCGCTCTGAATGC-3′	5′-GAGGGACTGAGCTGGACAACC-3′	66
DRD2 TaqIA RFLP	11q23.1	5′-CCGTCGACGGCTGGCCAAGTTGTCTA-3′	5′-CCGTCGACCCTTCCTGAGTGTCATCA-3′	53
COMT VAL158MET	22q11.2	5′-GGGCCTACTGTGGCTACTCA-3′	5′-GGCCCTTTTTCCAGGTCTG-3′	60
TPH A779C	11p15.3-14	5′-CCATTACTAAAGTATTATCACCCGATCAT-3′	5′-CAAGCCAATTTCTTGGGAGAAT-3′	61

**Table 2:** Allele frequency of the polymorphisms of the AVPR1A, SLC6A4, COMT, DRD2 and TPH1 analyzed in this study.

Gene	Allele	Freq.
AVPR1A	1	0.0041
AVR	2	0.0412
	3	0.1025
	4	0.3181
	5	0.4639
	6	0.0410
	7	0.0291
RS1	1	
	2	0.1046
	3	0.3645
	4	0.2357
	5	0.1102
	6	0.1133
	7	0.0122
	8	0.0554
	9	0.0041
RS3	1	0.0082
	2	0.0420
	3	0.0570
	4	0.1832
	5	0.2489
	6	0.1076
	7	0.1986
	8	0.0284
	9	0.0122
	10	0.0817
	11	0.0284
	12	0.0041
SLC6A4	L_A_	0.4863
5-HTTLPR	S	0.4028
	L_G_	0.1109
VNTR	9 repeats	0.0345
	10 repeats	0.4594
	12 repeats	0.5061
COMT	Val	0.4234
Val158Met	Met	0.5766
DRD2	A1	0.2455
TAQIA	A2	0.7545
TPH1	A	0.4327
A779C	C	0.5674

### SLC6A4

Three alleles of SLC6A4 promoter region 5-HTTLPR were genotyped combining the methods of Lesch et al. [47], Yonan et al. [48] and Rasmussen & Werge [49]. 5-HTTLPR PCR mixture (30 µl) contained 200 µM dNTP, 20 ng each primer (Table 1), 1× DyNAzyme EXT Buffer (50 mM Tris-HCl, 1,5 mM MgCl_2_, 15 mM (NH_4_)_2_SO_4_ and 0.1% Triton X100), 0.5 U DyNAzyme EXT DNA Polymerase (Finnzymes) and 25 ng DNA. The fragments obtained after digestion with NciI FastDigest (Fermentas) and 3% MetaPhor (Camprex Bio Science Rockland Inc., Rockland, Maine, USA) and agarose gel electrophoresis were short (S) (279, 127 and 62 bp), long variant L_A_ (339–342, 127 and 62 bp) and long variant L_G_ (174, 166, 127 and 62 bp). The allele frequency of the HTTLPR long L_A_ allele was 48%, the long L_G_ allele being 11% and the short allele S was 40%. Our frequencies of 5-HTTLPR alleles were in line with the previous studies reported by Hu et al. [13] (Table 2), whereas studies using biallelic S/L genotyping should be carefully interpreted [14]. Based on the evidence that both S and L_G_-alleles have a lowering effect on 5-HTT function [21], [22] statistical analyses were performed by combining these alleles as one allele.

Serotonin transporter (SLC6A4) microsatellite VNTR was analyzed by PCR (primers in Table 1) and run on an ABI 3730xl DNA Analyzer (Applied Biosystems, Foster City, CA, USA) and sized with GeneMapper 4.0 software (Applied Biosystems). Genotyping was successful in 92% of the subjects. For VNTR the 10 and 12 repeat alleles showed nearly equal distribution in the Finnish sample and the 9 repeat allele was present in >3% of the population (Table 2).

### TPH1, COMT and DRD2

TPH1 polymorphism A779C and COMT polymorphism Val158Met were analyzed by cycle sequencing with the Big Dye Terminator kit (version 3.1) supplied by ABI, and reactions were run on an ABI 3730 capillary sequencer according to manufacturer's instructions. Primers were designed according to Reuter et al. [2] The DRD2 TAQIA polymorphism was genotyped using the PCR-RFLP method described by Grandy et al. [37]. Genotyping was successful in 95%–96% of the subjects. The conditions are shown in Table 1 and allele frequencies in Table 2.

### Statistical analyses

A total of 484 individuals were included in the pedigrees and thus in the genetic analyses. Variance component linkage analysis (SOLAR) was used to calculate the heritability estimates [50] for all phenotypes, namely the test scores of the three musical aptitude tests KMT, SP, and ST, the combined score (denoted with COMB), as well as creativity and its subtypes composing, improvising and arranging. The combined music score (COMB) was computed as the sum of the separate scores of the three individual test results, where KMT music score was first scaled to the same range as the other music scores (ranging from 25 to 50 pts). An exact inverse normal transformation was subsequently performed on all the continuous phenotypes to ensure a normal distribution. Sex and age were routinely included as covariates in all analyses.

Genotype incompatibilities were searched with PedCheck [51]. PEDSTATS [52] was used to check the Hardy-Weinberg (HWE) equilibrium. No departure from HWE was observed for any of the markers. Marker allele frequencies were estimated by maximum likelihood in multigenerational families with SOLAR. IBD allele sharing probabilities were computed in a multipoint fashion using the software package Simwalk2.

For quantitative traits, family-based genetic association analyses were conducted using the program QTDT version 2.5.1 (http://www.sph.umich.edu/csg/abecasis/QTDT/) [53]. QTDT incorporates variance components methodology in the analysis of family data and includes exact estimation of p-values for analysis of small samples and non-normal data. Linkage and association are considered simultaneously and QTDT also enables taking covariates into consideration when evaluating the genetic association [53].

FBAT/HBAT (family-/haplotype-based association test) version 2.0.2c (http://www.biostat.harvard.edu/~fbat/default.html) was used to calculate family based association for creativity in music and its endophenotypes as well as all haplotypes; and also to ensure the results for quantitative phenotypes evaluated with QTDT. FBAT tests for association and linkage in pedigrees, using the general test statistic Z [54], which is based on a linear combination of offspring genotypes and traits. The null hypothesis is “no association and no linkage” between the marker locus and any trait-influencing locus. The alternative hypothesis states there is both association and linkage. FBAT handles pedigrees by breaking each pedigree into all possible nuclear families, and evaluating their contribution to the test statistic independently. A family is informative when it has a non-zero contribution to the FBAT statistic. Covariates can be included via estimation of a regression model in a separate statistical program; here we used SPSS. The covariates were sex and age for music test scores. The residuals of the model can then be used instead of the original traits Y_ij_ in the expression of the test statistic. FBAT additionally allows for multiallelic test for multiallelic markers and haplotypes, where the overall association and linkage of the marker/ markers is evaluated, circumventing the locus-wise multiple testing issues, but possibly losing some power. In addition, all results were controlled for multiple testing using permutation on FBAT/HBAT and QTDT. Here, we report both the multiallelic test results as well as single allelic tests. The minimum frequency of alleles and haplotypes to be taken into consideration was set at 0.05. Note, that while all possible two-marker haplotypes of RS1, RS3 and AVR were tested, the three-marker haplotypes RS3-RS1-AVR were not because HBAT cannot handle the high number (91) of different haplotypes with these three markers. In addition to the default, i.e. additive model of gene function, the dominant and recessive were also tested. However, no significant deviations from results by additive tests were observed; thus, these results are not included in the present article.

## Results

### Creativity in music is associated with high scores in musical aptitude tests

**Figure 2:**
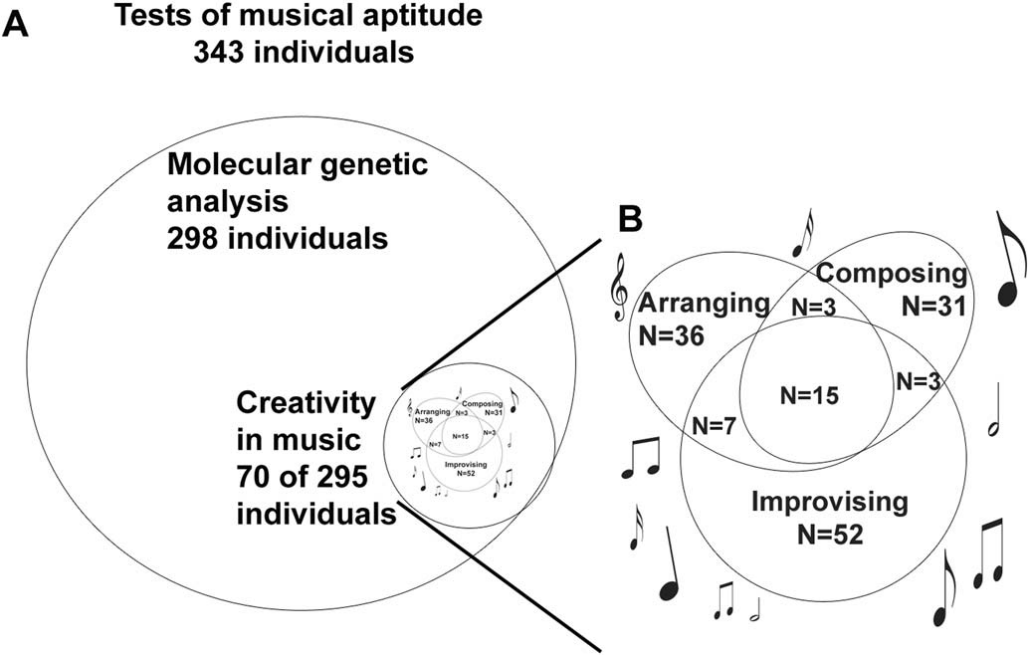
The participants of the study. A. Total of 343 individuals participated in the tests of musical aptitude and filled in the questionnaire, of them 298 gave DNA samples, and 70 reported creativity in music (composing, improvising and/or arranging). B. Subtypes of creativity in music. N = number of subjects.

**Figure 3:**
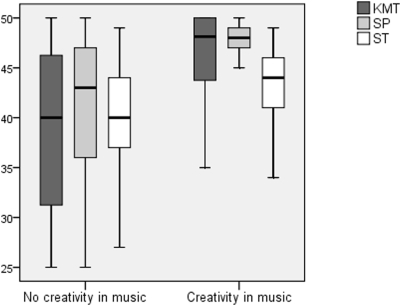
The relationship between music test score (KMT, SP and ST) and creativity in music.

From 295 participants (86% of the material) 70 (24%) reported creativity in music (Fig. 2). A total of 31 subjects (10.5%) reported that they composed (mean age 29.2 years), 36 (12.2%) arranged (mean age 31.5 years), and 52 (17.6%) improvised music (mean age 30.6 years). 15 subjects (5.1%) engaged in all of the three activities. In our study, creative functions (here total creativity in music) were associated with high scores in music tests (Fig. 3). SP, ST and KMT were all statistically significantly higher in individuals with creative functions in music compared to non-creative ones (Mann-Whitney KMT p<0.001, SP p<0.001, ST p = 0.001, COMB p = 0.001).

### Heritability estimates

**Figure 4:**
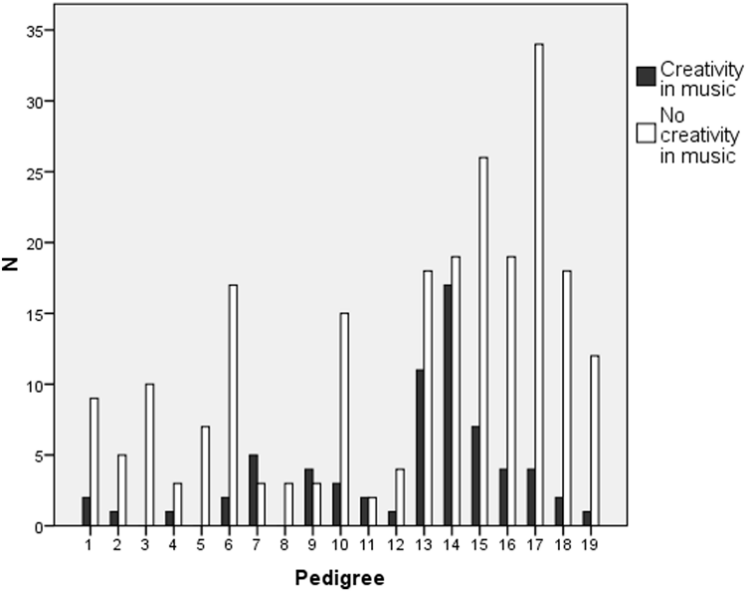
The distribution of self-reported creativity in music in the 19 pedigrees.

The heritability of the music test scores in the new families 16–19 (Fig. 1) was significant for KMT and SP (Table 3), and overall in agreement with the heritability estimates from our previous study [40]. Intriguingly, we obtained relatively high estimates of heritability for creativity in music, too (Table 3). Detailed analysis of the pedigrees showed that creative functions were enriched in pedigrees 7, 9, 13 and 14 whereas in families 3 and 8 no creative functions were reported (Figure 4).

### Individual genetic effects

**Table 3:** Heritability estimates of the music test scores and creativity in music.

Phenotype	Families 1–19 (Fam. 16–19)	
	h^2^	p
Karma Music Test (KMT)	0.39 (0.57)	1×10^−7^
Seashore pitch (SP)	0.52 (0.66)	7.4×10^−12^
Seashore time (ST)	0.10 (0.20)	0.10
Combined (COMB)	0.44 (0.68)	1.6×10^−9^
Creativity in music	0.84	2.8×10^−5^
- Composing	0.40	8.5×10^−3^
- Arranging	0.46	7.7×10^−3^
- Improvising	0.62	9.9×10^−4^

**Table 4:** The results of FBAT/HBAT analyses a. for the music test scores (quantitative traits).

Trait	Gene	Polymorphism	Allele(s)	Freq./informative fam#	p	Corrected p
KMT	AVPR1A	AVR	6	0.040/17	0.00732	NS
		AVR and RS1	Overall			0.02751
		AVR and RS1	4 and 4	0.103/20		0.02751
		RS1 and RS3	Overall			0.00612
		RS1 and RS3	4 and 4	0.042/11	0.0167	0.0192
		**RS1 and RS3**	**4 and 5**	**0.103/21**	**0.000807**	**0.00002**
		**RS1 and RS3**	**5 and 4**	**0.063/10**		**0.00032**
	SLC6A4	VNTR 5-HTTLPR	12 repeats and LA	0.171/33		0.0115
SP	AVPR1A	RS3	4	0.198/45	0.0267	NS
		RS1+RS3	4 and 5	0.103/21	0.0261	0.0072
		RS1+RS3	5 and 4	0.063/10	0.0268	0.0154
ST	AVPR1A	AVR and RS1	5 and 4	0.149/28	0.0038	0.00184
		AVR and RS3	4 and 4	0.052/11	0.0352	0.00534
COMB	AVPR1A	AVR and RS1	Overall		0.0043	0.04546
		AVR and RS1	5 and 4	0.149/28	0.0083	0.00402
		RS1 and RS3	Overall		0.0104	0.06491
		**RS1 and RS3**	**4 and 5**	**0.103/21**	**0.0056**	**0.00060**
		**RS1 and RS3**	**5 and 4**	**0.063/10**	**0.0018**	**0.00064**

The most significant findings are shown in bold.

#### AVPR1A

Haplotype RS1+RS3 showed strongest association with KMT (the most prominent haplotype 4 5, corrected p = 0.00002; 5 4 haplotype, corrected p = 0.00032) as well as with combined music test scores (COMB) (4 5 haplotype, corrected p = 0.00060; 5 4 haplotype corrected p = 0.00064; overall, corrected p = 0.0649) (Table 4). Haplotypes RS1+RS3 showed overall association with KMT (corrected p = 0.00612). An allelic association was shown between KMT and the AVR microsatellite allele 6 (uncorrected p = 0.0073; corrected p = NS) (Table 4). RS1+RS3 haplotype showed also some association with SP (corrected p = 0.0072). Finally, combined music test scores (COMB) showed overall association with AVR+RS1 haplotype (p-value 0.0043; corrected p = 0.0455), specifically with AVR+RS1 5 4 (corrected p = 0.0040). Some evidence for association between arranging and AVR+RS1 4 3 haplotype was obtained (corrected p = 0.00392). The results using QTDT were not remarkable (data not shown).

#### SLC6A4

There was only very weak evidence for association with KMT and haplotype VNTR (12 repeats) +5-HTTLPR (L_A_) (corrected p = 0.0115) (Table 4). The results of creative functions in music were not remarkable and no significant overall p-values were found considering this locus (data not shown).

#### TPH1, COMTVal158Met and DRD2 TAQIA polymorphism

FBAT analysis of TPH1 A799C showed suggestive overall association on composing (uncorrected p = 0.0089; corrected p = 0.0107) (Table 5). No significant main effects were found on the TPH1 A799C polymorphism in relation to musical aptitude test scores using QTDT or FBAT (data not shown). Using QTDT COMT SNP Val158Met showed weak evidence for overall association with SP (corrected p = 0.0526) (data not shown). Additionally some association with improvising was seen with Val-allele (corrected p = 0.012). No significant main effects with music test scores or creative functions and DRD2 were seen.

**Table 5:** The results of FBAT/HBAT analyses for the creativity in music (categorical traits).

Trait	Gene	Polymorphism	Allele(s)	Informative fam#	p	Corrected p
Composing	TPH1	A779C	A	40	0.00887	0.01066
Improvising	COMT	Val158Met	Val	42	0.01437	0.0120
Arranging	AVPR1A	AVR+RS1	4 and 3	16	0.0379	0.00392

The most significant findings are shown in bold.

## Discussion

Humans have always had a passion for the highest levels of creativity, which may have an affect on continuous development of the human civilization. Music culture and the music industry are dependent on artistic creativity. We show for the first time two lines of evidence for the role of a genetic liability for creative functions in music in this study: composing, improvising and arranging are practiced by subjects with high music test scores that contain substantial genetic component [40, this study] and composing, improvising and arranging occur in some families but not in others that have got high scores in music tests.

We show here that the *AVPR1A* haplotypes are associated with auditory structuring ability in music (KMT). The strongest effect was obtained with RS1+RS3 haplotype. In addition, Seashore's test for time (ST) and for pitch (SP) showed suggestive association with AVPR1-haplotypes. Associations with AVPR1A-haplotypes were replicated with combined music test scores (COMB). Interestingly, several overlapping loci were found in the genome-wide scan of musical aptitude using KMT, ST and COMB [40] suggesting that these relatively different tests and their combination may have a common biological background. The KMT is devised to measure auditory structuring by using small, abstract sound patterns that are repeated to form hierarchic structures. The subject's task is to detect structural changes in these patterns, i.e., changes in the order or number of the tones. In contrast, SP and ST subtests consist of pair-wise comparisons of the physical properties of sound and are used to measure simple sensory capacities, such as abilities to detect small differences in tone pitch or length. However, we cannot exclude the risks that the music tests measure partially same traits thus containing a risk of multiple testing in causing overlapping results. This is specifically a case, if the tests also measure the use of sound in social contacts (see below).

Interestingly, *AVPR1A* has been known to modulate social cognition and behavior (see the recent review by Donaldson and Young [55]) making it a strong candidate gene for music perception and production. Several features in perceiving and practicing music, a multi-sensory process, are closely related to attachment [56]. Based on animal studies Darwin proposed in 1871 that singing is used to attract the opposite sex. Furthermore, lullabies are implied to attach infant to a parent and singing or playing music together may add group cohesion [57]. Thus, it is justified to hypothesize that music perception and creativity in music are linked to the same phenotypic spectrum of human cognitive social skills, like human bonding [13] and altruism [17] both associated with AVPR1A. It is of notice that both altruism (also called pathological trusting), and intense interest towards music and relatively sparse language skills are the characteristic features of Williams-Beuren syndrome (WBS), a neurodevelopmental syndrome with elfin facial features, supravalvular aortic stenosis, hypercalcemia and scoliosis [55], [58]. AVPR1A is also associated with autism, an opposite phenotype with poor social communication skills [14], [46], [59].

Independently, AVPR1A showed some association with arranging. When arranging music the ability to gestalt musical emotions, melodies, and rhythms is essential. Improvising music is the inter-subjective co-ordination of musical acts with other musicians or/and between a musician and the listeners [60].

Musician's motivation for creative functions is greatly emotional and connected to social communication [60], [61]. Here, tentative evidence for association of TPH1 A779 –allele was obtained with composing. Intriguingly, in the study of Reuter et al. [2] the A allele was related to figural and numeric creativity, skills that are required in composing. In addition, musicians have been found to attain significantly higher spatial test scores than non-musicians [62]. The spatial abilities may be related to the ability to read and memorize notes. Furthermore, numeric creativity may be important to musicians because it may be connected to the ability to perceive and understand rhythms. Investigating the lately discovered TPH2 [63] would also be important in the near future, but at this point we preferred the polymorphisms used in the studies of Bachner-Melman et al. [23], Yirmiya et al. [46] and Reuter et al. [2].

From the genes previously studied as candidate genes for human creativity [2] the COMT Val158Met is weakly associated, in our study, with both pitch recognition (SP) and improvising. The results are in line with the phenotype data above where creative activity was only found in the presence of good pitch recognition and auditory structuring ability. DRD2 TAQIA was suggestively related to Seashores test score of time perception (QTDT-analysis p = 0.0192, uncorrected). Recent data has shown that A1 allele is linked to courtship [64], presenting the emotional view of DRD2. In the study of Reuter et al. [2] DRD2 A1 allele was related to higher verbal creativity.) The evolutionary background of music and language can be speculated here based on partially overlapping brain regions in brain PET studies [65]. Improvising music is the inter-subjective co-ordination of musical acts with other musicians or/and between a musician and the listeners [66], a tool for social communication. As creative individuals in music are scarce, even in musicians' families, the endophenotype groups remain small. Here the nominally significant findings may be considered as in the context of low power to detect the relatively weak association expected at a marker in a complex genetic trait like musical aptitude [40]. However, we cannot exclude the role of high music test scores to the allele/haplotype associations obtained with creative functions in this study.

In this study the web-based questionnaire, technique which is becoming increasingly common in the various fields of the human research [67], [68], was used to define creativity in music. The pros, including time and money saving, as well as the possible cons, like poor diversity (age, sex, and education), lack of motivation, non-serious responses and dishonesty, additional drop-outs, anonymity, and multiple submissions [69], of the method were considered. The use of the Internet did not affect the diversity of the data or the number of drop-outs because alternative answering opportunities were given as an option (paper-based questionnaire and parents answering on behalf of children). The families participating in the study include musicians, do so on a voluntary basis, and they are highly motivated to further the study, minimizing non-serious responses and dishonesty. The cons of our study may be that data about the creativity in music is based on self-report and individuals with creativity in music are quite rare, while the pros are that our pedigrees contain more individuals with musical aptitude and further creativity in music, than average pedigrees.

Creativity and divergent thinking are sometimes considered as divisions of intelligence, suggesting creativity has a strong genetic basis (and as normal distribution) in population, as intelligence. Various candidate genes related to intelligence, neuronal development and neurotransmission, have been proposed, but the genetic basis of cognitive ability is still under debate [35]. The results of our recent genome wide scan of musical aptitude showed overlapping loci with dyslexia [40] referring to the hypothesis about common evolutionary background of music and language. Dopaminergic and serotoninergic systems have been shown to have a role in cognitive functioning. Although creativity is part of human cognitive function, there are difficulties to define creativity and to measure it. In our dataset creativity in music was seen mainly in individuals with high music test scores. The subtypes of creativity in music (composing, improvising and arranging) were practiced from all three together to only one of them. Perceiving, processing and creating music takes place at multiple sites and elicits different functions of the brain. Creativity is a multifactorial genetic trait involving a complex network made up of a number of genes [70]. The association of AVPRIA, COMT and TPH1 polymorphisms with the different subtypes of creativity in music may imply the emotional elements required.
